# Comparison of Phacoemulsification and Aspiration Parameters in Cataract Surgery: Metal Tip vs. Hybrid Tip

**DOI:** 10.3390/bioengineering11121195

**Published:** 2024-11-26

**Authors:** Kazuo Ichikawa, Kei Ichikawa, Seiji Tokiwa, Yuki Sato, Tomoyuki Miyazaki, Yoshiki Tanaka, Naoki Yamamoto

**Affiliations:** 1 Chukyo Eye Clinic, Nagoya, Aichi 456-0032, Japan; kei@chukyogroup.jp (K.I.); stokiwa@chukyomedical.co.jp (S.T.); y.sato@chukyo-eyeclinic.jp (Y.S.); tmiyazaki@chukyo-eyeclinic.jp (T.M.); ytanaka@chukyomedical.co.jp (Y.T.); 2 General Aoyama Hospital, Toyokawa, Aichi 441-0103, Japan; 3 Center for Society-Academia Collaboration, Research Promotion Headquarters, Fujita Health University, Toyoake, Aichi 470-1192, Japan; naokiy@fujita-hu.ac.jp; 4 International Center for Cell and Gene Therapy, Research Promotion Headquarters, Fujita Health University, Toyoake, Aichi 470-1192, Japan

**Keywords:** INTREPID Balanced Tip, INTREPID Hybrid Tip, total ultrasound time, cumulative dissipated energy, total aspiration time, total estimated fluid aspirated, corneal endothelial cell reduction, lens capsule rupture

## Abstract

Various tips are available for phacoemulsification in cataract surgery. Evidence-based data can inform ophthalmologists, especially inexperienced ones, on tip selection. We retrospectively evaluated the energy efficiency and other parameters of two ultrasonic phacoemulsification and aspiration tips across different nuclear hardness grades in 342 cataract patients (342 eyes) with nuclear hardness grades II to IV. Surgical procedures, ultrasound settings, and instrumentation were standardized. All surgeries were performed by one experienced doctor. We compared the metal INTREPID^®^ Balanced Tip (M-tip) with the INTREPID^®^ Hybrid Tip (P-tip), which has a polymer coating. The M-tip required significantly less total ultrasound time and cumulative dissipated energy (CDE) than the P-tip for grades III and IV, while the P-tip had a shorter aspiration time and less estimated fluid aspirated for grade II. No differences in corneal endothelial cell loss were observed. Lens rupture rates were low: 0.47% for the M-tip and 0.78% for the P-tip. Multiple regression analysis showed that CDE increased with nuclear hardness. These findings suggest that the M-tip is efficient for harder lenses, while the P-tip may be advantageous for softer lenses, informing optimal tip selection in cataract surgery. Further research is suggested to elucidate their clinical significance.

## 1. Introduction

Phacoemulsification (phaco) and aspiration, pioneered by Dr. Charles Kelman in 1967 [1], are now the primary methods used in cataract surgery worldwide. Over time, a range of innovative techniques—including phaco tips and sleeves—and agents such as viscoelastic materials have significantly improved the effectiveness, outcomes, and safety of cataract surgery [2]. Beyond the primary goal of removing the opacified lens and restoring visual acuity, recent advances in cataract surgery techniques, devices, and intraocular lenses aim to meet patients’ demands for high-quality vision and postoperative satisfaction.

A serious complication in cataract surgery is corneal endothelial cell damage. These cells are essential for regulating corneal hydration, and their damage during surgery can lead to corneal edema (bullous keratopathy) [3]. Monitoring during cataract surgery is vital to prevent such complications, and various factors are incorporated into surgical devices [4,5]. Cumulative dissipated energy (CDE), a measure of the ultrasound (US) energy used to remove a cataract, is an important parameter in the Alcon Infiniti^®^ Vision System (Alcon Laboratories, Inc., Fort Worth, TX, USA) [6]. Lower CDE during surgery indicates less US energy used, reducing the impact on corneal endothelial cells [7,8,9,10].

In cataract surgery, a tip is attached to the end of a phaco handpiece, vibrating longitudinally with US to fragment the cloudy lens [2]. While metal (medical-grade titanium alloy) is the traditional material for these tips, a high-strength polymer-overmolded tip has recently been developed [11]. Previous studies have reported various combinations of new surgical equipment and handpieces [4,12], demonstrating reduced US energy requirement for lens emulsification [13,14,15,16,17,18,19,20,21].

This study compares the performance of two phaco tips across different cataract grades (II, III, IV) under uniform surgical conditions, with all procedures performed by the same surgeon. We analyzed parameters from these surgeries, including the change in corneal endothelial cell count pre- and post-surgery, to provide insights into the performance of the two tips.

## 2. Materials and Methods

### 2.1. Phacoemulsification Tips

**Figure 1:**
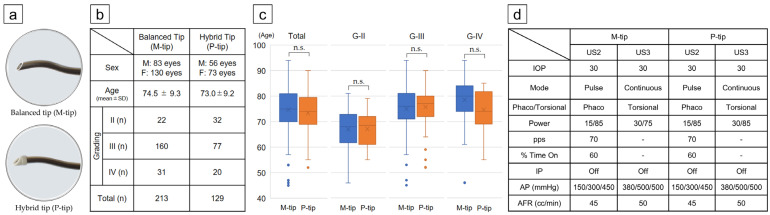
Breakdown of patient and equipment conditions for cataract surgery. (**a**) Appearance of M-tip and P-tip. (**b**) Breakdown of patients treated with M-tip and P-tip. (**c**) No significant differences in patient age were observed across all cases and by Emery–Little classification grade. (**d**) Ultrasonic power and irrigation settings were standardized. Abbreviations: AFR, aspiration flow rate; AP, aspiration pressure; F, female; IOP, intraocular pressure; M, male; pps, pulse per second; US, ultrasound; n.s., not significant.

Surgeries were grouped by the two phaco tips used: the metal INTREPID^®^ Balanced Tip (M-tip, Alcon Laboratories, Inc.), designed to minimize movement near the incision while generating a large lateral amplitude at the tip for efficient operation, and an INTREPID^®^ Hybrid Tip (P-tip, Alcon Laboratories, Inc.), which features a rounded edge and high-strength polymeric material at the tip of an M-tip to reduce the risk of capsule rupture while delivering the same amplitude force (Figure 1a). Both tips were used with the CENTURION^®^ ACTIVE SENTRY^®^ handpiece (Alcon Laboratories, Inc.), which includes a perfusion pressure sensor [22] conventionally located on the equipment, but now positioned on the handpiece to detect fluctuations in intraocular pressure more accurately.

### 2.2. Cataract Patients

The study included 342 eyes of 342 patients who underwent cataract surgery at Chukyo Eye Clinic between June 2021 and June 2022. For patients who had surgery in both eyes, the eye operated on first was enrolled in the study, as none of the patients had cataract grades that differed significantly between eyes. The patients were randomly assigned to two groups: the M-tip group, comprising 213 eyes (83 males, 130 females, age 74.5 ± 9.3 yrs.), and the P-tip group, comprising 129 eyes (56 males, 73 females, age 73.0 ± 9.2 yrs.) (Figure 1b). Note that the group sizes were uneven because the P-tip was marketed later than the M-tip; the priority during the study period was maintaining uniform surgical conditions. The nuclear hardness of the cataracts was graded using the Emery–Little classification system [23], with only grades II (G-II), III (G-III), and IV (G-IV) included. Eyes with other complications (e.g., ocular diseases other than cataracts, such as corneal or retinal diseases and glaucoma), intraoperative iris hypotony syndrome, zonular weakness, false dropout syndrome, high myopia, or nuclear hardness greater than G-IV (e.g., mature cataract is not amenable to phacoemulsification) were excluded (Figure 1c). 

This retrospective study was approved by the Chukyo Eye Clinic Ethics Committee (No. 20220301-01) and adhered to the tenets of the Declaration of Helsinki. Due to the retrospective design, the Ethics Committee approved an opt-out method for including patient data to maintain anonymity instead of requiring written informed consent, which had been obtained for the original operations.

### 2.3. Surgical Procedure

Intraoperative data were analyzed to compare the performance of the M-tip and P-tip. All surgeries were performed by the same highly experienced cataract surgeon, with (US) power and irrigation settings uniformly configured (Figure 1d). The procedure began with US2 (without torsional setting) until the point of lens nucleation, then switched to US3 (with torsional setting) for nucleation. The P-Tip is constructed from a polymer material attached to the M-Tip. When the phaco setting is identical, the stroke length varies due to the polymer material, which is an important factor in nucleation. Different stroke lengths result in variations in nucleation performance. With the power of the cataract surgery device (Centurion^®^ Vision System (Alcon Laboratories, Inc.)) set at 100%, the stroke length was 192 μm for the M-tip and 165 μm for the P-tip [4]. Therefore, based on a previous report, the US3 power was set to 75 for the M-tip and 85 for the P-tip, making the stroke lengths nearly identical [4].

Anesthesia procedures were performed in the same manner as follows: ophthalmic anesthesia was performed with 0.4% Benoxil^®^ ophthalmic solution (Santen Pharmaceutical Co., Ltd., Osaka city, Osaka, Japan), and 0.2–0.3 mL of 1% Xylocaine^®^ Injection solution (Sandoz Pharma K.K.) was injected through the main opening port using a Healon needle.

The instruments were standardized: the main incision was made using a Beaver^®^ Xstar^®^ KOJO Slit Knife (3.2 mm, 45 degrees, single bevel, Beaver-Visitec International, Inc., Waltham, MA, USA). A 0.9 mm high-infusion sleeve (Alcon Laboratories, Inc.) was used to accommodate fluid dynamics influenced by different sizes, potentially affecting the surgical technique. The crystalline lens was fragmented using the Spatula Nucleus Dividing and Separating CHUKYO (Inami Co., Ltd., Bunkyo-ku, Tokyo, Japan) instrument, and viscoelastic material was chosen based on nucleus hardness, with a soft shell used when applicable. Other US surgical techniques that do not directly impact the surgery were based on the patient’s condition.

### 2.4. Comparison Criteria

The parameters compared for phaco cataract surgery were (1) total US time, (2) CDE, (3) total aspiration time, (4) total estimated fluid aspirated, (5) corneal endothelial cell reduction (pre- to post-surgery), and (6) rate of lens capsule rupture. CDE, representing the total US energy delivered during phacoemulsification, was measured in units used by the Centurion^®^ Vision System (Alcon Laboratories, Inc.). CDE, accounting for the power and time of both longitudinal and torsional US modes, was calculated as follows:

CDE = (phaco time × average phaco power) + (torsional time × 0.4* × average torsional power). *0.4 = Coefficient representing the approximate reduction in heat dissipation at the incision site compared to conventional phacoemulsification.

A specular microscope (CEM-530 PARACENTRAL^®^, NIDEK Co., Ltd., Gamagori city, Aichi, Japan) was used to examine change in corneal endothelial cell count based on pre- and post-operative evaluations.

### 2.5. Statistical Analysis

Data are presented as the mean ± standard deviation (SD) and were analyzed using Welch’s *t*-test and analysis of co-variance (ANCOVA). Multiple regression analysis was conducted to examine correlations between nuclear hardness classification and each parameter. A chi-square test was used to assess lens capsule rupture rates. Statistical analyses were performed using the Statistical Package for Social Science (SPSS) Statistics 24 version 27 (IBM, New York, NY, USA). Power analysis calculations were performed using the software application known as “easy R”, EZR version 1.68.

## 3. Results

In this retrospective study, no significant differences were found between the M-tip and P-tip groups in terms of patient demographics or cataract grades based on the Emery–Little classification (Figure 1).

### 3.1. Total US Time

**Figure 2:**
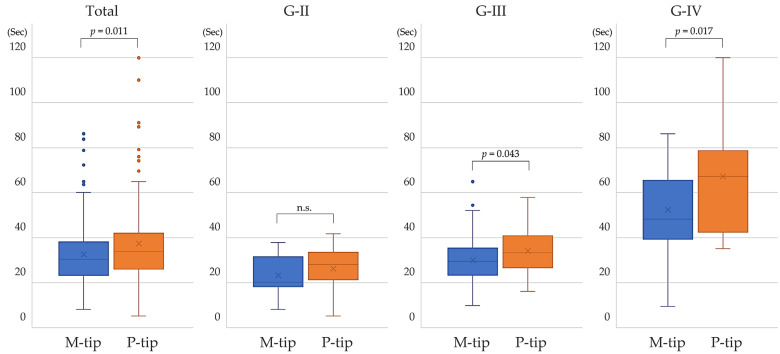
Comparison of total ultrasound time between M-tip and P-tip by nuclear hardness. The M-tip required significantly less time than the P-tip in all cases for G-III and G-IV. There was no significant difference for G-II. Abbreviations: n.s., not significant.

The total US time was significantly shorter with the M-tip across all cases (*p* = 0.011). When analyzed by nuclear hardness classification, the M-tip also showed significantly shorter US time for G-III (*p* = 0.043) and G-IV (*p* = 0.017), but not for G-II (*p* = 0.254) (Figure 2). A shorter US time may lead to less intraocular heat generation, which could be beneficial for endothelial cell preservation.

### 3.2. CDE

**Figure 3:**
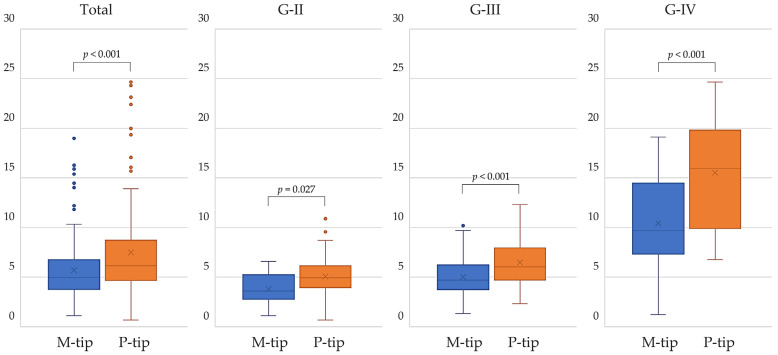
Comparison of CDE between M-tip and P-tip by nuclear hardness. The M-tip showed significantly lower CDE than the P-tip in all cases and across all nuclear hardness levels.

CDE values were significantly lower with the M-tip (*p* < 0.001) compared to the P-tip across all cases (*p* = 0.001). When analyzed by nuclear hardness classification, the M-tip also had significantly lower CDE for G-II (*p* = 0.027), G-III (*p* < 0.001), and G-IV (*p* = 0.001) (Figure 3). Lower CDE means less exposure to ultrasound energy in the eye, which may lead to fewer complications such as corneal edema and endothelial cell damage.

### 3.3. Total Aspiration Time

**Figure 4:**
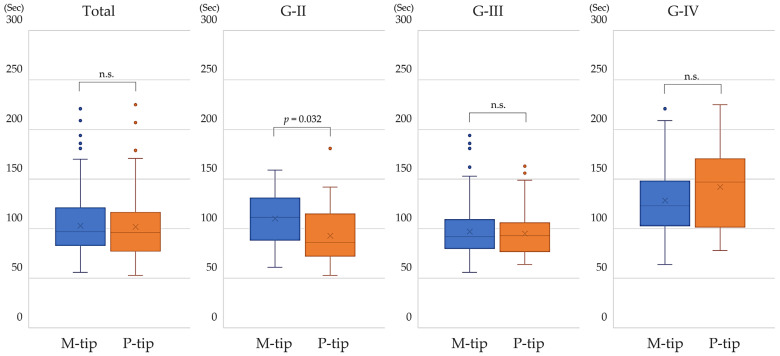
Comparison of total aspiration time between M-tip and P-tip by nuclear hardness. The P-tip showed a significantly shorter aspiration time in eyes with G-II nuclear hardness. No significant differences were found for G-III, G-IV, or across all eyes regardless of nuclear hardness.

Total aspiration time was defined as the cumulative duration during which the phacoemulsification equipment actively aspirated fluid and lens material, as tracked by the surgical system during the procedure. Overall, there was no significant difference in total aspiration time between the M-tip and P-tip groups. However, for G-II, the P-tip demonstrated a slightly but significantly shorter aspiration time (*p* = 0.032). The difference in total aspiration time due to the different tips does not significantly affect the overall operative time (Figure 4).

### 3.4. Total Estimated Fluid Aspirated

**Figure 5:**
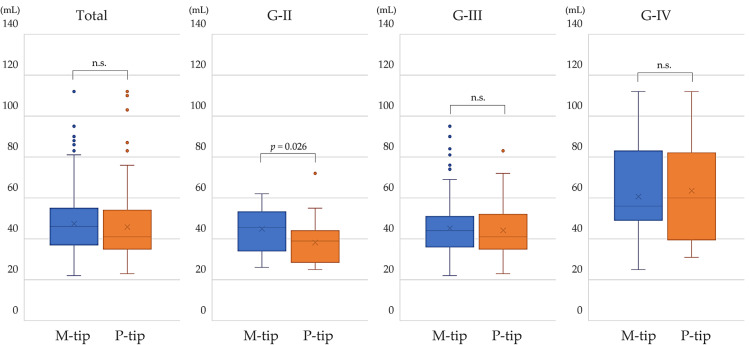
Comparison of total estimated fluid aspirated between M-tip and P-tip by nuclear hardness. The P-tip was associated with a significantly lower amount of fluid aspirated in G-II. No significant differences were observed in G-III, G-IV, or across all cases or nuclear hardnesses. Abbreviations: n.s., not significant.

There was no significant overall difference in total estimated fluid aspirated between the groups. However, the P-tip showed a slight but significant reduction in fluid aspirated for G-II (*p* = 0.026). Averaged over all cases, the P-tip aspirated 3.0% less fluid compared to the M-tip (Figure 5).

### 3.5. Corneal Endothelial Reduction Rate

**Figure 6:**
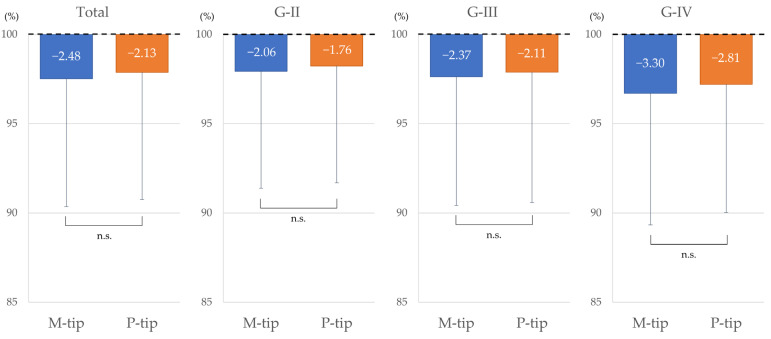
Comparison of corneal endothelial cell reduction rate between M-tip and P-tip by nuclear hardness. No significant differences were observed across all cases or nuclear hardness levels. Abbreviations: n.s., not significant.

There was no significant difference in the corneal endothelial cell reduction rate. Tips were equally safe for corneal endothelial cells, but the P-tip was safer in cases with low cell density (Figure 6).

### 3.6. Statistical Tests Analysis

Multiple regression analysis revealed that CDE was significantly correlated with increasing nuclear hardness in both the M-tip (*p* = 0.0053) and P-tip (*p* = 0.0232) groups. Additionally, total aspiration time showed a non-significant but increasing trend with increasing nuclear hardness (Table 1 and Table 2).

### 3.7. Rates of Lens Capsule Rupture

Lens capsule rupture occurred in 1 out of 213 cases (0.47%) in the M-tip group and 1 out of 129 cases (0.78%) in the P-tip group. The rupture in the M-tip group occurred in a G-II cases, while in the P-tip group, it occurred in a G-III case. A chi-square test showed no significant associations (*p* = 0.7097).

## 4. Discussion

In this study, we compared two different phacoemulsification tips (M-tip and P-tip) used by the same surgeon with extensive cataract surgery experience, under standardized surgical equipment and conditions. The surgical outcomes, including corneal endothelial cell reduction, were analyzed across different cataract grades.

Previous studies have evaluated various combinations of handpieces and tips. For instance, a study of 116 eyes comparing the 45° Balanced Tip with the 45° Kelman Tip found that the Balanced Tip required significantly less total US time, CDE, and balanced salt solution (BSS) usage [24]. Another study involving 150 eyes compared the 30° mini-flared 12° bent tip (Ozil), the 45° mini-flared 22° bent tip (Kelman), and the mini-flared 45° Balanced Tip. The Ozil group showed higher phaco power and mean torsional amplitude, with significantly greater BSS usage compared to the Kelman group, but no significant difference was found between the Balanced Tip group and the other groups [25]. Furthermore, in a study of 248 eyes comparing the 45° Balanced Tip and the 45° Kelman Tip, it was again demonstrated that the Balanced Tip had lower total US time, CDE, aspiration time, and BSS usage [12]. Another study of 168 cataract surgeries randomly assigned to two combinations, namely the Active Sentry handpiece with the Hybrid Tip and the Centurion Ozil handpiece with the Balanced Tip [4], found significantly lower CDE and torsional amplitude with the Active Sentry and Balanced Tip combination, although there were no significant differences were observed in total aspiration time or estimated fluid aspirated. Furthermore, the Centurion Ozil handpiece and Balanced Tip combination was associated with posterior capsule rupture in two eyes and iris damage in one eye [4].

While some studies have shown a positive correlation between CDE levels and corneal endothelial cell loss, others have found no significant relationship [26,27,28,29,30,31]. In our study, the P-tip showed significantly higher CDE than the M-tip, but there was no significant difference in corneal endothelial cell reduction between the tips. Although we observed a slight trend toward greater corneal endothelial preservation with the M-tip, it was not clinically significant. Previous findings indicated that a higher nuclear sclerotic cataract grade, according to the Lens Opacities Classification System III (LOCS III), was associated with higher CDE, and multivariate analysis showed significant associations between higher CDE and NS grade ≥ 2 [32].

Previous reports and our present report cannot be compared in general because of the difference in tips and cataract surgery devices. M-tips are made of metal and have a sharp tip, which is more destructive to the lens. On the other hand, the P-tip, which is a polymer coated M-tip, has a slightly rounded tip, which is less destructive than the M-tip but has improved safety by making the lens capsule rupture.

In our study of age-related nuclear cataracts, Scheimpflug imaging (Pentacam) was used, and cataract grading was performed using LOCS III. A different study found a positive correlation between Scheimpflug measurements and LOCS III grading scores. Measurement by the former correlated with CDE, torsional amplitude, and time [33]. In that study, CDE was more strongly correlated with Scheimpflug-measured lens nuclear density (*r* = 0.797) than with LOCS III NO or nuclear color scores (*r* = 0.614 and *r* = 0.637, respectively). We graded nucleus hardness based on the Emery–Little classification. The multiple regression analysis results indicated a significant correlation between CDE and increased cataract grade for both the M-tip (*p* = 0.0053) and P-tip (*p* = 0.0232).

Under the standardized conditions of this study, the M-tip demonstrated greater efficacy in lens destruction, requiring less US time and resulting in lower CDE compared to the P-tip. The slightly shorter total aspiration time and lower estimated fluid aspirated with the P-tip in G-II cataracts may be attributed to the increased cross-sectional area due to the polymer coating on the P-tip, which could influence aspiration dynamics. The adsorption power depends on the difference between atmospheric and suction pressure and the adsorption surface area of the tip (Supplemental Figure S1). Additionally, the white polymer coating on the P-tip enhances tip visibility [34]. As a result, the surgeon’s impression is that the P-tip also makes it easier to aspirate the crushed lens nucleus and surrounding cortex. In particular, if the tip is not brought close to the periphery of the lens capsule, but held in the center of the lens, the surgeon’s impression is that the lens contents can be safely aspirated. On the other hand, the M-tip is more useful for hard lenses because it is metallic and has a pointed tip compared to the P-tip. There was no difference in postoperative visual acuity results between the two types of tips. No significant age (stratified by 10 years) or sex difference was found between M-tip and P-tip groups in measurement data collected at the time of surgery. As cataract grade increased, the difference between the tips became less pronounced, likely due to factors such as water flow dynamics, vortex generation at the tip during aspiration, and the hardness of the lens material being aspirated. The clinical significance of total aspiration time is that it reflects the efficiency and effectiveness of the procedure. A shorter aspiration time suggests quicker removal of lens material, potentially reducing the overall duration of the surgery, minimizing exposure to ultrasonic energy, and lowering the risk of complications such as corneal endothelial cell loss or other mechanical damage to the eye.

In an experimental study using porcine crystalline lenses, no significant differences in efficiency, ultrasonic mode time, or CDE were observed between the Centurion Vision System with a 30° bevel metal Balanced Tip (similar to the M-tip) and a hybrid polymer tip (similar to the P-tip) [35]. In our own previous experiments, we evaluated the risk of capsule rupture in porcine lenses with both tips under different power modes (longitudinal, torsional, and combined) and suction pressures (0 and 200 mmHg) [34]. The M-tip consistently showed lower US power and fewer capsule ruptures compared to the P-tip, with no significant differences within the M-tip across power modes. In contrast, the P-tip showed the least likelihood of capsule rupture in torsional mode.

In this clinical study, no significant difference in lens capsule rupture rates was found between the two tips. Since this finding may be due to the surgeon’s extensive experience, further studies including surgeon experience as an independent variable are warranted. However, it has been reported that recent cataract surgery requires less energy to emulsify opacified lenses than in the past due to fewer cases presenting with high-grade or scleral nuclei [4,13,14,15,16,17,35]. In addition to completely removing the material of opaque lenses, smaller incision sizes and advanced IOLs significantly reduce the risk of postoperative corneal astigmatism, and postoperative visual acuity is better due to refractive correction [4]. This indicates that cataract and refractive surgery is increasingly being performed not simply to restore vision, but also to improve the quality of vision. Also, for surgeons with less experience of performing cataract surgery, equipment designed to offer a higher level of patient safety is preferable. Thus, the use of hybrid tips may prove to be safer in the long term.

This study has some limitations. We focused on cataracts graded G-II to G-IV using the Emery–Little classification, so the findings may not be generalizable to cases outside this range. Additionally, the results may have differed if another classification system was used, necessitating reclassification. The higher number of G-III cases compared to G-II and G-IV suggests that statistically significant differences were more likely to be detected in G-III.

## 5. Conclusions

This study compared the surgical parameters, corneal endothelial cell reduction rate, and lens capsule rupture rate in phacoemulsification cataract surgery performed by one highly experienced surgeon using two different tips, the M-tip and the P-tip. The M-tip was associated with shorter total ultrasound time and lower cumulative dissipated energy across all cataract grades studied, while the P-tip showed slightly shorter total aspiration time and lower estimated fluid aspirated in G-II cases. No significant differences were found in corneal endothelial cell reduction rate or lens capsule rupture rate between the tips. For novice surgeons embarking on a career in cataract surgery, the hybrid tip may prove to be the safer choice. Although some differences in cataract grade were statistically significant, they were not substantial in terms of surgical outcomes. In other words, there was little difference in operative time, suggesting that both tips are safe for surgery by ophthalmologists with extensive experience in cataract surgery.

## Figures and Tables

**Table 1:** Results of multiple regression analysis for the correlation between nuclear hardness classification and various parameters, as well as reduction rates of corneal endothelial cells in M-tip and P-tip groups. Abbreviations: US, ultrasound; CDE, cumulative dissipated energy.

M-tip	β	S.E.	*t*	*p*-value	95% CI		
Total US time	0.0043	0.0068	0.6289	0.5301	−0.0091	—	0.0177
CDE	0.0819	0.0291	2.8174	0.0053	0.0246	—	0.1391
Total aspiration time	−0.0015	0.0011	−1.3136	0.1904	−0.0037	—	0.0007
Total estimated fluid aspirated	0.0002	0.0026	0.0955	0.9240	−0.0049	—	0.0053
Reduction rates of cells	−0.0058	0.0041	−1.3922	0.1654	−0.0140	—	0.0024
P-tip	β	S.E.	*t*	*p*-value	95% CI		
Total US time	0.0096	0.0052	1.8578	0.0656	−0.0006	—	0.0199
CDE	0.0462	0.0201	2.2987	0.0232	0.0064	—	0.0860
Total aspiration time	-0.00004	0.0021	−0.0195	0.9845	−0.0043	—	0.0042
Total estimated fluid aspirated	0.0030	0.0043	0.6927	0.4898	−0.0055	—	0.0115
Reduction rates of cells	−0.0030	0.0063	−0.4727	0.6373	−0.0154	—	0.0095

**Table 2:** Statistical tests compared M-tip and P-tip for 5 items in cataract surgery. Results of Welch’s *t*-test, analysis of co-variance (ANCOVA) and power analysis. Significant differences shown in bold.

	Grade	Welch’s *t*-Test	Analysis of Co-Variance (ANCOVA)		Power Analysis
			Nuclear Grade as Covariate	Compared to Chip	
Total US time	G-II	*p* = 0.254	***p* < 0.001**	***p* < 0.001**	0.709
	G-III	***p* = 0.043**			
	G-IV	***p* = 0.017**			
	Total	***p* = 0.011**			
CDE	G-II	***p* = 0.027**	***p* < 0.001**	***p* < 0.001**	0.988
	G-III	***p* < 0.001**			
	G-IV	***p* < 0.001**			
	Total	***p* < 0.001**			
Total aspiration time	G-II	***p* = 0.032**	***p* < 0.001**	*p* = 0.654	0.026
	G-III	*p* = 0.714			
	G-IV	*p* = 0.250			
	Total	*p* = 0.764			
Total estimated fluid aspirated	G-II	***p* = 0.026**	***p* < 0.001**	*p* = 0.918	0.108
	G-III	*p* = 0.570			
	G-IV	*p* = 0.654			
	Total	*p* = 0.359			
Corneal endothelial cell reduction rate	G-II	*p* = 0.878	*p* = 0.433	*p* = 0.756	1.000
	G-III	*p* = 0.812			
	G-IV	*p* = 0.830			
	Total	*p* = 0.688			

## Data Availability

Dataset available on request from the authors.

## Supplementary Materials

The following supporting information can be downloaded at: https://www.mdpi.com/article/10.3390/bioengineering11121195/s1, Figure S1: M-tip and P-tip images and adsorption power formula. The adsorption area of M-tip (A) and that of P-tip (A’). The adsorption area at the tip of P-tip is slightly larger to the extent that the tip of M-tip is coated with a polymer. W = Theoretical adsorption power. P0 = Atmospheric pressure. P = Suction pressure. A = M-tip adsorption surface area. A’ = P-tip adsorption surface area. T = Force due to atmospheric pressure.
