# Supplementary material for: Comparison of Phacoemulsification and Aspiration Parameters in Cataract Surgery: Metal Tip vs. Hybrid Tip
Source: Bioengineering (Basel). 2024 Nov 26;11(12):1195. doi: 10.3390/bioengineering11121195 (PMC11727584; doi:10.3390/bioengineering11121195)
Supplement: Supplementary file 1 [file bioengineering-11-01195-s001.zip › bioengineering-3294703-supplementary.pptx]

## Slide 1
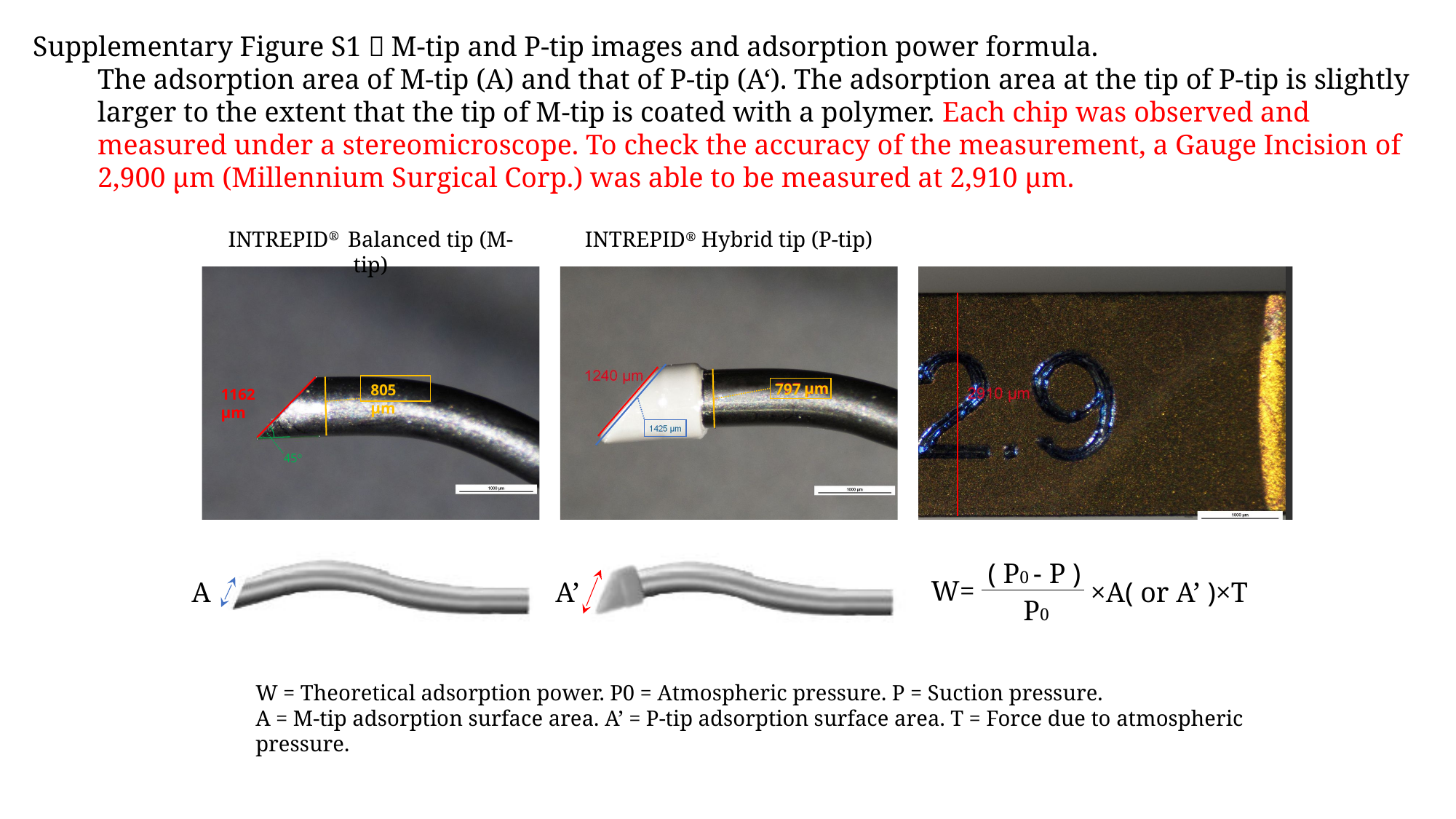

Supplementary Figure S1：M-tip and P-tip images and adsorption power formula.
The adsorption area of M-tip (A) and that of P-tip (A‘). The adsorption area at the tip of P-tip is slightly larger to the extent that the tip of M-tip is coated with a polymer. Each chip was observed and measured under a stereomicroscope. To check the accuracy of the measurement, a Gauge Incision of 2,900 μm (Millennium Surgical Corp.) was able to be measured at 2,910 μm.
INTREPID® Balanced tip (M-tip)
INTREPID® Hybrid tip (P-tip)
µm
797
805 µm
1162 µm
45°
( P0 - P )
W=
×A( or A’ )×T
A
A’
P0
W = Theoretical adsorption power. P0 = Atmospheric pressure. P = Suction pressure.
A = M-tip adsorption surface area. A’ = P-tip adsorption surface area. T = Force due to atmospheric pressure.
